# Acupoints as Neuromodulation Interfaces: Translating Classical Body Maps into Neural Circuit Science

**DOI:** 10.3390/brainsci16080835

**Published:** 2026-08-06

**Authors:** Beomku Kang, Da-Eun Yoon, Yeonhee Ryu, Younbyoung Chae

**Affiliations:** 1 Department of Meridian and Acupoints, College of Korean Medicine, Kyung Hee University, Seoul 02447, Republic of Korea; beomkukang@khu.ac.kr (B.K.); yoonda05@khu.ac.kr (D.-E.Y.); 2 KM Fundamental Research Division, Korea Institute of Oriental Medicine, Daejeon 34054, Republic of Korea; yhryu@kiom.re.kr

**Keywords:** acupuncture, neuromodulation, acupoints, stimulation–engagement–response model, sacral neural circuits, biomarker-guided stimulation

## Abstract

**Highlights:**

**What are the main findings?**

- Acupoints can be understood as relational body-map nodes linking stimulation, sensation, symptoms, and physiological responses.
- The Baliao region is used to illustrate how a candidate neuromodulation interface involving sacral and pelvic regulatory circuits could be prospectively tested.

**What are the implications of the main findings?**

- Acupuncture research should integrate multiple dimensions of stimulation, target engagement, and physiological response, together with anatomical and individual variability.
- Imaging, physiological measures, and patient stratification may enable personalized and adaptive neuromodulation therapy.

**Abstract:**

**Background/Objectives:** Modern acupuncture research commonly operationalizes acupoints as standardized sites for needling or electrical stimulation. Although this convention has improved reproducibility, it may obscure how classical acupoint and meridian systems have historically organized relationships among intervention sites, sensory phenomena, symptom patterns, and therapeutic responses. This article proposes an operational framework for examining selected acupoint regions as candidate neuromodulation interfaces. It reframes acupoint and meridian systems as relational body maps and operationalizes this perspective through a multidimensional stimulation–engagement–response model that distinguishes intervention features, target engagement, and downstream functional outcomes while accounting for individual context. **Methods:** The framework was developed to organize existing evidence, identify unresolved causal links, and generate prospective experimental tests, rather than to establish neural or physiological mechanisms that have already been demonstrated. The Baliao region, comprising bilateral BL31–BL34 over the posterior sacral foramina, was selected as the primary case because classical localization and pelvic symptom associations can be examined alongside sacral anatomy and neurophysiology within a single region. Relevant clinical and preclinical findings were evaluated in relation to potential neural targets, physiological engagement, functional outcomes, and alternative explanations. **Results:** Clinical and preclinical evidence suggests possible engagement of sacral afferent, spinal, autonomic, and pelvic-floor regulatory circuits. However, the evidence remains indirect, heterogeneous, and insufficient to establish equivalence with implantable sacral neuromodulation, a unique BL31–BL34 mechanism, or absolute point specificity. The framework clarifies what current evidence supports, identifies unresolved mechanistic links and alternative explanations, and proposes prospective strategies for testing target engagement and physiological relevance. **Conclusions:** This approach provides a staged research agenda for moving from descriptive associations toward prospective tests of parameter-dependent target engagement, physiological relevance, and, ultimately, biomarker-guided neuromodulation.

## 1. Introduction

Acupoints are frequently investigated as discrete anatomical loci, with emphasis on their correspondence to nerves, vessels, connective tissue planes, or other anatomically identifiable structures [1,2]. Such work is valuable, but an exclusively substrate-oriented approach risks reducing classical body maps to a retrospective search for anatomical correlates [3,4]. A complementary approach is to treat acupoint–meridian systems as relational body maps: historically accumulated and stabilized models that organize relationships among intervention sites, sensory phenomena, symptom topographies, and therapeutic responses [5]. This formulation neither requires traditional concepts such as meridians to correspond to contemporary physiological pathways nor reduces selection of acupoints to target-based, fixed neuroanatomical loci. Instead, it treats classical body maps as sources of experimentally tractable hypotheses about tissue recruitment, afferent signaling, autonomic regulation, and patient-specific therapeutic response. Within this framework, selected acupoint regions can then be examined as neuromodulation target candidates through which defined intervention sites are translated into testable models of neural, autonomic, and somatic regulation.

Neuromodulation broadly refers to interventions that alter nerve activity by delivering electrical or other targeted stimuli to neural structures, often within the peripheral nervous system [6]. Within this broader field, electrically based neuromodulatory therapies are increasingly conceptualized as electroceuticals, which use electrical impulses rather than pharmacological agents to modulate physiological function [7]. In the application of such therapies, target selection is critical since it directly shapes the nature of the treatment. The term “neuromodulation interface” generally describes the physical interface through which energy is transferred from a device to a specific neural structure. Here, we redefine the term to encompass an anatomical–functional region on our body surface in which stimulation parameters, tissue context, sensory recruitment, and physiological responses can be systematically related. In this context, neuromodulation interfaces become testable zones of interaction among peripheral afferents, connective and muscular tissues, spinal circuits, autonomic regulation, and supraspinal networks [8]. This approach may aid in translating classical acupoint knowledge into anatomically grounded and physiologically tractable research questions without reducing it to traditional doctrine or fixed neuroanatomical mapping.

This perspective is particularly relevant to nerve–organ interactions, because acupoint stimulation can be conceptualized not only as local tissue stimulation but also as a means by which peripheral afferent input modulates organ-specific autonomic and spinal regulatory circuits [9]. To move beyond a general comparison between classical acupoint theory and contemporary biomedical science, we focus on a region where historical clinical knowledge and modern neurophysiology plausibly converge. The Baliao points comprise four paired acupoints—BL31, BL32, BL33, and BL34—located bilaterally over the posterior sacral foramina. They have long been used in East Asian medicine for disorders involving the lower back, pelvis, urinary and reproductive systems, and anorectal region [10,11]. Anatomically, this region can be examined as a candidate sacral anatomical–functional interface through which pathways involved in bladder, bowel, pelvic floor, and pelvic pain regulation might be investigated.

Previous publications from our group have conceptualized acupoints as probabilistic or relational body maps and proposed spatial and double-dissociation approaches for examining acupoint specificity [2,5,12]. The present framework further advances the concept by addressing a deeper level of analysis. Rather than focusing primarily on how acupoint locations are represented or whether different acupoints produce dissociable effects, it specifies how a candidate body region can be translated into a testable neuromodulation model by separating intervention features, target engagement, and downstream function and by defining the experimental relationships required among them. The Baliao case is used to illustrate this translational and hypothesis-generating process rather than to retrospectively validate a specific mechanism.

This article has two objectives: first, to develop a multidimensional stimulation–engagement–response framework for translating selected regions within relational body maps into testable neuromodulation interfaces, and second, to apply this framework to the Baliao region by evaluating the current evidence, identifying unresolved mechanistic links, and outlining a staged research agenda for prospective validation. By integrating classical textual evidence, anatomical localization, clinical and preclinical findings, and biomarker-guided stimulation strategies, we outline a mechanism-oriented research agenda for acupuncture-informed neuromodulation.

## 2. From Relational Body Maps to Testable Neuromodulation Interfaces

An acupoint-derived neuromodulation interface is best defined not as a fixed point or a presumed neural target but as a reproducible relationship among a spatially defined body region, a specified stimulation configuration, and a measurable physiological response [13]. Historical descriptions and recurring clinical associations may identify a region as a candidate for investigation, but they cannot by themselves establish such an interface. The central question is whether stimulation delivered to a defined region, with specified depth, trajectory, and parameters, engages identifiable tissues or afferent pathways and produces responses relevant to a prespecified organ system or regulatory circuit.

This definition comprises three experimentally separable but conceptually linked components. The spatial component concerns how the candidate region is defined and how anatomical variation influences the tissues accessible from that region [2,14]. The stimulation component concerns parameter-dependent recruitment: changes in stimulation depth, trajectory, intensity, frequency, or pulse width should produce corresponding changes in sensory, muscular, neural, or reflex responses [15]. The functional component concerns whether such engagement is associated with mechanism-aligned measures of organ function, autonomic regulation, reflex excitability, or neural activity. The interface is therefore defined by experimentally demonstrable relationships among stimulation location, stimulation parameters, target engagement, and physiologically relevant outcomes, rather than by any one of these features in isolation.

Evidence for a proposed interface is strengthened when these relationships are examined across a coordinated experimental program. Anatomical and imaging studies can characterize the tissues accessible from a candidate region; parameter-mapping studies can determine how stimulation configurations influence proximal sensory, muscular, neural, or reflex responses; and physiological studies can test whether those responses are associated with prespecified organ- or circuit-related outcomes. Where feasible, measuring adjacent links within the same study can strengthen inference, but no single experiment is expected to validate the entire model. Informative comparison conditions and independent replication are needed to determine whether the proposed relationships are reproducible. Once such relationships have been established, anatomical or physiological responses may also be used to optimize stimulation, stratify responders, or predict clinical outcomes as later translational extensions of the model [16,17,18].

The framework is intended to be applicable across body regions, but its validity cannot be assumed to generalize automatically from one acupoint region to another. Each candidate region must be evaluated in relation to its own anatomical accessibility, stimulation-dependent tissue recruitment, and measurable functional domain. PC6, ST36, SP6, and auricular vagus-related regions illustrate how distinct interface models might be developed around median nerve afferents, lower-limb somato-autonomic pathways, pelvic-visceral regulation, or auricular vagal–trigeminal afferent systems [12,19,20]. These examples are presented as candidates for region-specific investigation rather than as established acupoint–nerve correspondences (Table 1).

Baliao was selected because historical localization, foraminal anatomy, pelvic symptom associations, and measurable sacral physiology converge within a relatively bounded region, allowing the proposed framework to be illustrated and prospectively tested. Sacral neuromodulation also provides an established biomedical comparator, although it remains a distinct intervention. Baliao is therefore used as an illustrative test case for evaluating the interface concept. The following sections examine its historical and anatomical definition, the current clinical and experimental evidence, and the links that remain to be tested prospectively.

## 3. The Baliao Region as a Primary Case Model

The name Baliao, commonly translated as “eight crevices,” provides a particularly informative test case for this framework. Its historical localization is not merely proportional or symbolic but anchored in palpable sacral anatomy, as reflected in the term liao, which denotes a cleft or foramen-like depression. This anatomical anchoring does not reduce Baliao to a single sacral nerve or foraminal structure. Rather, it identifies a regional interface in which osseous morphology, pelvic symptom topography, sensory access, and sacral regulatory physiology can be experimentally related. CT-based three-dimensional reconstruction studies have shown substantial individual variation in sacral foraminal morphology, supporting the use of anatomical reference points for accurate localization of BL31-BL34 [21]. Within this framework, anatomy-based localization may provide a plausible route for recruiting sacral afferent pathways and pelvic regulatory circuits, although recruitment is likely to vary with needle trajectory, depth, stimulation intensity, and individual morphology.

The Huangdi Neijing provides an early textual anchor for this regional interpretation. In the Suwen chapter “Gukong lun,” Baliao is discussed in relation to low back pain with restricted movement and radiation toward the genital region and is localized to the lumbosacral spaces. This passage should not be read as evidence of modern sacral neuroanatomical knowledge. Its relevance is instead relational: it links a lumbosacral intervention field to a recurring clinical pattern involving local pain, restricted movement, and pelvic or genital symptoms.

**Figure 1:**
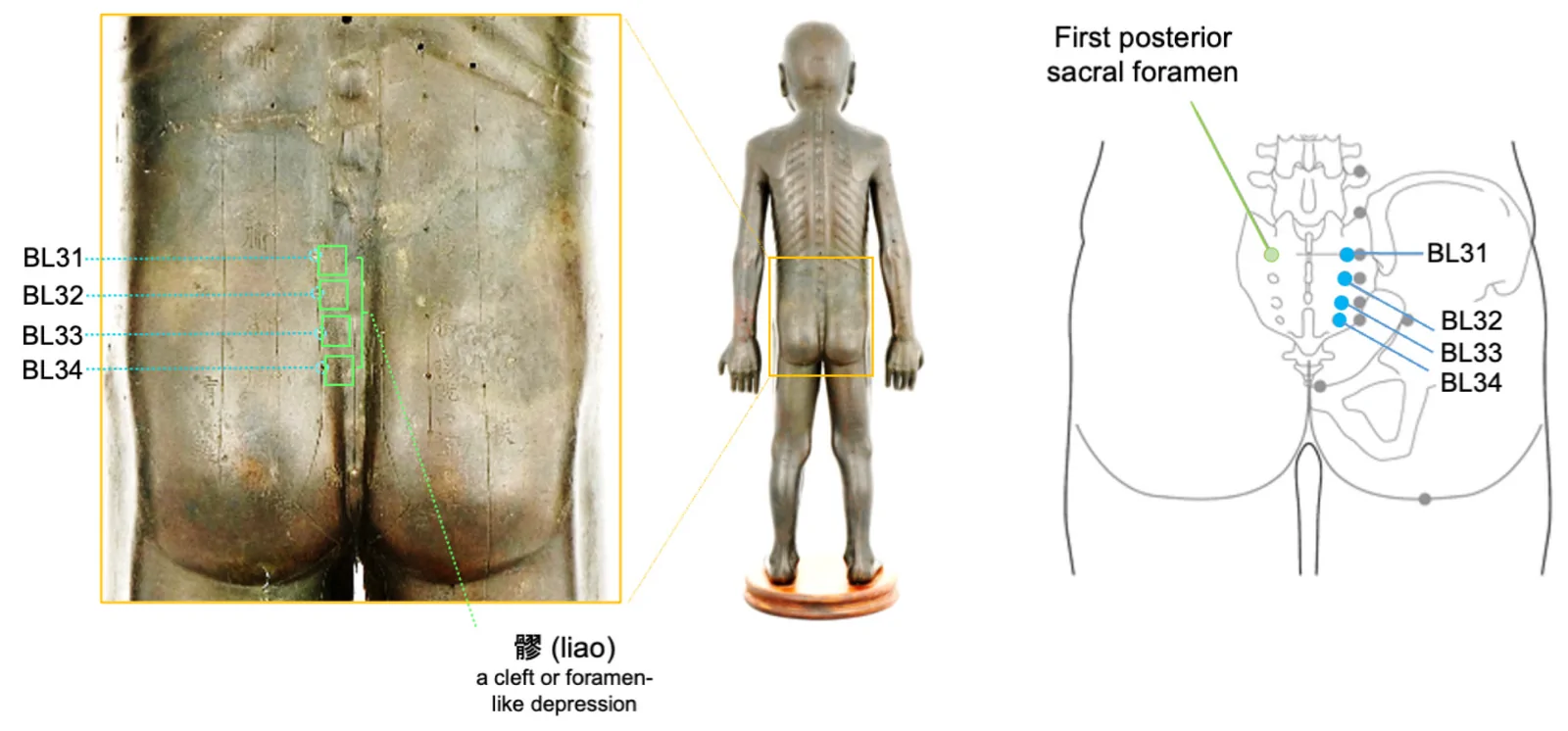
Classical and anatomical representation of the Baliao points. The posterior pelvic region of a classical acupuncture bronze figure shows BL31-BL34 arranged vertically in the sacral area. The enlarged view highlights their traditional surface localization, while the schematic diagram illustrates the WHO-standardized locations of BL31-BL34 (marked in blue dots) in relation to the first through fourth posterior sacral foramina. The term liao (髎), meaning a cleft or foramen-like depression, reflects the anatomical rationale embedded in the nomenclature of the Baliao points. Grey dots indicate traditional acupoints near the sacral area. Sources: Image of the classical acupuncture bronze figure modified from the collection of the National Palace Museum of Korea. Chimgeum Dongin (鍼金銅人), 1741, accession no. Changdeok 26757; www.gogung.go.kr. Right schematic diagram adapted from the World Health Organization Regional Office for the Western Pacific, WHO Standard Acupuncture Point Locations in the Western Pacific Region. All images were modified by the authors for clarity and illustrative purposes.

Material evidence from later periods also supports the view of acupoints as embodied spatial knowledge. The eighteenth-century Korean acupuncture bronze figure Chimgeum Dongin (1741), preserved at the National Palace Museum of Korea, displays meridian lines and acupoints engraved across the body surface, including the posterior trunk and lumbosacral region. As a model likely used for acupuncture training or examination, it indicates that acupoints were not merely textual or symbolic entities but standardized body sites that could be taught, localized, and assessed on the human form. This embodied tradition is consistent with modern standardization: in the World Health Organization (WHO) standard acupuncture point locations, BL31-BL34 are explicitly located in the first through fourth posterior sacral foramina, respectively. This material tradition provides a historical bridge for interpreting the Baliao region as a clinically stabilized sacral acupoint field (Figure 1).

Together, these features make the sacral acupoint region a methodologically informative case for examining the present framework. Its value lies in the convergence of classical localization, foraminal anatomy, pelvic symptom clustering, and contemporary pelvic neurophysiology. Implantable sacral neuromodulation, most commonly targeting the S3 nerve root, serves as an informative comparator rather than an equivalent model. Baliao-region electroacupuncture differs fundamentally in invasiveness, spatial precision, waveform control, dosing logic, and durability of effect [22]. The relevant comparison therefore concerns partial territorial and functional overlap within sacral systems governing bladder, bowel, pelvic floor, and pelvic pain regulation [23]. Accordingly, Baliao is framed here as a classically defined sacral intervention field, distinct from sacral nerve stimulation but suitable for study as a neuromodulation interface.

## 4. Current Evidence for Baliao as a Candidate Neuromodulation Interface

Following the framework outlined above, the current evidence can be evaluated according to whether stimulation of the anatomically defined Baliao region is linked to measurable engagement of sacral pathways and physiologically relevant pelvic outcomes. Existing studies address different parts of this relationship, but few directly connect stimulation location and parameters, proximal target engagement, and downstream physiological or clinical responses.

The evidence reviewed below supports different levels of inference. Clinical trials provide evidence of functional effects under specific treatment protocols but generally do not identify the tissues or pathways responsible for those effects. Preclinical studies provide biological plausibility for sacral sensory and spinal modulation but do not directly establish target engagement in the human Baliao region. Neither evidence stream currently demonstrates the complete causal chain from a defined stimulation configuration through proximal target engagement to downstream physiological or clinical function.

### 4.1. Clinical Evidence for Functional Relevance

Clinical studies suggest that electrical stimulation of the lumbosacral acupoint region can influence selected pelvic-floor and lower urinary tract outcomes. In women with stress urinary incontinence, electroacupuncture involving BL33 and BL35 produced greater reductions in urinary leakage than sham treatment [24]. Additional trials have reported potential benefits in pure stress urinary incontinence and when electroacupuncture was added to pelvic-floor muscle training [25,26]. More recently, bilateral electroacupuncture involving the Baliao region accelerated early continence recovery following radical prostatectomy compared with sham stimulation [27].

These findings support the functional relevance of lumbosacral stimulation under the tested protocols. However, they do not establish that the observed effects depend specifically on BL31–BL34 or identify the tissues and neural pathways through which the responses were produced. The studies differ in point selection, needling depth and trajectory, electrical parameters, treatment dose, comparator design, and clinical phenotype. Some protocols also include adjacent points outside the four Baliao locations, further limiting attribution of the effects to a standardized Baliao configuration.

Thus, the clinical evidence supports the protocol-specific functional relevance of lumbosacral stimulation but remains non-mechanistic with respect to Baliao-specific tissue recruitment and causal pathways.

Randomized studies of implantable sacral neuromodulation have demonstrated benefits for overactive bladder and severe fecal incontinence [28,29,30]. These findings do not constitute evidence for Baliao stimulation itself, but they demonstrate that modulation of sacral regulatory systems can alter bladder and bowel function. Implantable sacral neuromodulation therefore provides a physiological and therapeutic comparator rather than an equivalent intervention. The convergence lies primarily in the pelvic functions potentially modulated, whereas the interventions differ substantially in tissue access, spatial precision, waveform control, treatment dose, and durability of stimulation.

### 4.2. Experimental Evidence for Sacral Target Engagement

Preclinical studies provide evidence that stimulation of the sacral acupoint region can alter bladder-related sensory and spinal activity under controlled experimental conditions. In a rat model of detrusor hyperreflexia, electroacupuncture at BL32 inhibited bladder overactivity and reduced c-fos expression in the sacral spinal cord, suggesting modulation of bladder-related afferent input or spinal reflex processing [31]. A related animal and human study implicated TRPV1/ATP signaling in electroacupuncture-mediated regulation of bladder dysfunction, reporting changes in TRPV1 expression and urinary ATP concentrations together with improved recovery of spontaneous voiding after acute or postoperative urinary retention [32].

These studies support the biological plausibility of sacral sensory and spinal modulation, but they do not fully identify the pathway linking peripheral stimulation to pelvic-organ regulation. The measured molecular and spinal responses may reflect engagement of several interacting tissues and afferent systems. Their application to the human Baliao region is also limited by differences in anatomy, point localization, stimulation depth, electrical dose, and outcome measurement between experimental models and clinical protocols. The findings are therefore most informative as evidence of the biological plausibility that sacral-region stimulation can influence bladder-related afferent and spinal processing.

### 4.3. Integrated Assessment and Unresolved Links

Taken together, the available evidence supports Baliao as a candidate, rather than an established neuromodulation interface. The region has a relatively well-defined historical and anatomical basis, and clinical studies suggest that lumbosacral electroacupuncture can influence selected urinary and pelvic-floor outcomes. Preclinical observations further indicate that sacral-region stimulation can modify bladder-related sensory and spinal processes. However, the central links proposed by the interface model remain incompletely characterized.

In particular, existing studies rarely determine which tissues and afferent pathways are recruited by a specified Baliao stimulation configuration, whether recruitment varies systematically with depth, trajectory, intensity, or electrical parameters, and whether proximal target engagement predicts downstream physiological or clinical responses. Candidate pathways may include cutaneous and muscular afferents, sacral dorsal rami, and downstream pudendal-related, spinal, autonomic, or pelvic-floor circuits. Supraspinal modulation of interoception, pain, placebo effects, and autonomic control is also plausible but has not been directly demonstrated for Baliao-region stimulation.

The current evidence also does not clearly distinguish responses associated with the standardized Baliao locations from broader effects of lumbosacral stimulation. Nearby stimulation sites may recruit overlapping tissues, meaning that either positive or null comparisons between locations require interpretation in relation to the structures actually engaged. Future studies should therefore compare competing tissue- and circuit-level models by relating variations in stimulation location and parameters to proximal target-engagement measures and prespecified pelvic physiological outcomes. The principal objective is not to demonstrate absolute point specificity but to determine which stimulation configurations produce reproducible and functionally relevant neuromodulatory responses.

## 5. From Point Selection to a Multidimensional Stimulation–Engagement-Response Model

If acupoints are understood as elements within relational body maps, the experimental question extends beyond which named point should be selected [33]. The relevant object of investigation is the broader stimulation–response relationship through which intervention at a defined body region produces measurable physiological effects. Fixed protocols centered primarily on point selection have contributed to standardization and clinical reproducibility, but they may treat biologically distinct interventions as equivalent when they share the same acupoint label. A more informative model should consider how stimulation location, anatomical access, modality, depth, trajectory, electrical parameters, treatment dose, elicited responses, recruited tissues and pathways, and the physiological state of the individual interact to shape the resulting response.

The Baliao region illustrates this multidimensional relationship. Stimulation at the same named point may engage different tissues depending on posterior sacral foraminal anatomy, needle trajectory and depth, stimulation intensity, and the use of manual or electrical stimulation. Conversely, stimulation at adjacent surface locations may recruit overlapping sacral, muscular, or sensory structures. Manual acupuncture, electroacupuncture, percutaneous electrical stimulation, and implantable sacral neuromodulation may therefore operate within partially overlapping sacral regulatory territories, but they should not be treated as equivalent interventions. They differ in anatomical access, invasiveness, spatial precision, waveform control, stimulation duration, reversibility, and durability of effect. Their comparison is most informative when these differences are related explicitly to the tissues and pathways recruited and to the physiological responses observed.

Elicited sensory, muscular, neural, and reflex responses form an additional part of this relationship. Sensations reported during stimulation, visible or electromyographic muscle activity, reflex responses, and early changes in organ function may provide information about tissue recruitment and target engagement. These responses should not automatically be interpreted as evidence of a specific mechanism, but neither should they be treated only as procedural details or nonspecific by-products. Their value depends on whether they vary systematically with stimulation features, can be reproduced, and are associated with prespecified downstream physiological outcomes. The same stimulation protocol may also produce different responses across individuals because of variation in anatomy, baseline physiological state, clinical phenotype, and sensory responsiveness. Because these responses occur close to the site and timing of stimulation, they are particularly useful for initially testing whether changes in stimulation parameters produce corresponding changes in tissue or pathway recruitment.

**Figure 2:**
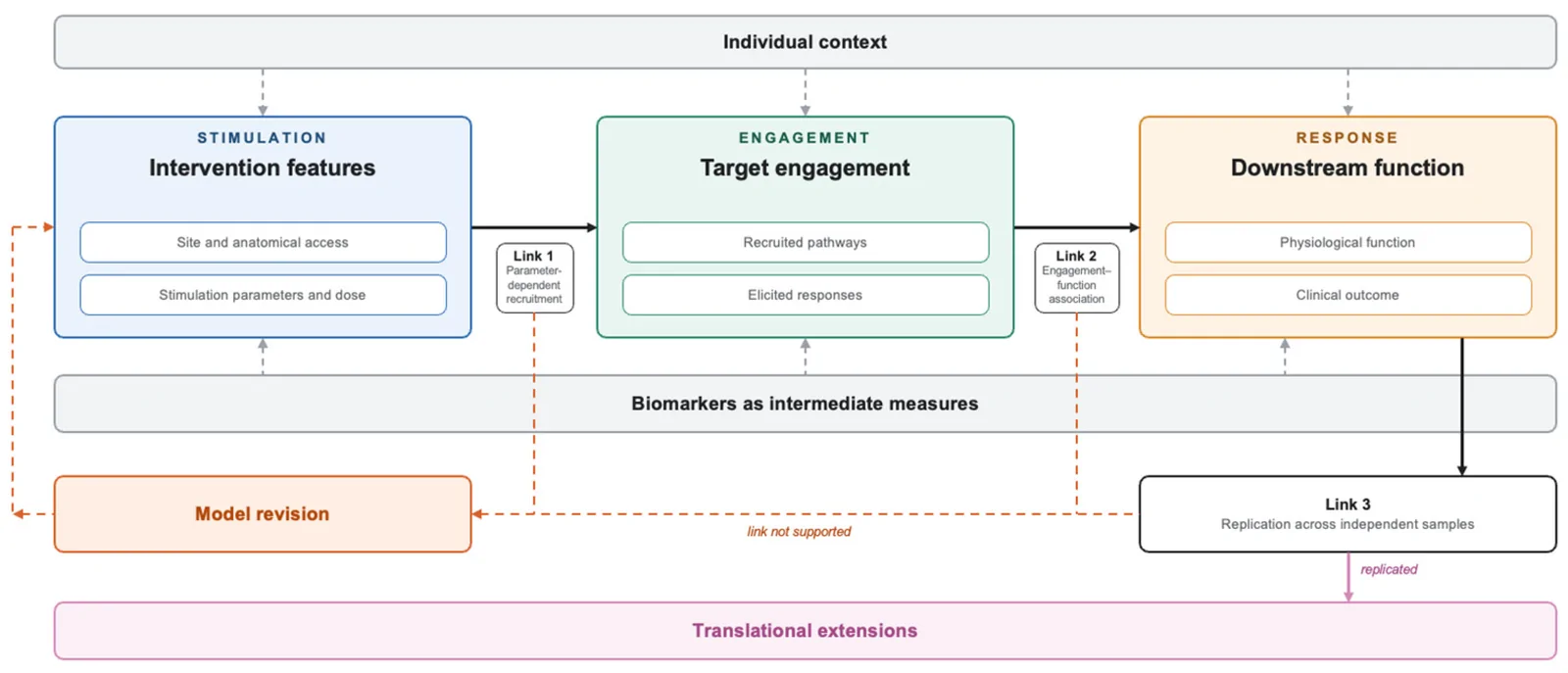
A multidimensional stimulation–engagement–response model for acupoint-derived neuromodulation interfaces. Stimulation (intervention features: what was delivered), engagement (target engagement: what was recruited), and response (downstream function: what changed) are treated as related but experimentally distinguishable layers. Intervention features comprise the stimulated site and its anatomical access, together with stimulation parameters and dose. Within the engagement layer, recruited pathways are inferred, whereas elicited responses—sensation quality and distribution, pelvic-floor electromyography, and reflex and evoked responses—are measured during or immediately after stimulation. Within the response layer, physiological function explains the process through which a benefit may have occurred, whereas clinical outcome demonstrates benefit under a particular protocol. Individual context modifies each layer, and biomarkers act as intermediate measures connecting what was delivered, what was recruited, and what changed. The interface is defined not by any component in isolation but by three demonstrable relationships: parameter-dependent recruitment (Link 1), engagement–function association (Link 2), and replication across independent samples (Link 3). Failure to support any link (dashed return path) requires modification of the recruitment model, restriction of its functional scope, or rejection of the model for that indication. Translational extensions follow only from replicated relationships. The schema is region-general and is illustrated here for the Baliao region.

This perspective changes how acupuncture-informed neuromodulation should be designed and interpreted. Rather than comparing point labels or fixed protocols alone, studies should distinguish intervention variables, individual modifiers, proximal engagement responses, and downstream functional outcomes. Biomarkers serve within this model as intermediate measures that can help connect what was delivered, what was recruited, and what physiological change followed. No single study is expected to capture every component, but coordinated studies should progressively establish reproducible links across the stimulation–engagement–response chain. Once these relationships have been demonstrated, they may support parameter optimization, responder stratification, and eventually biomarker-guided or adaptive stimulation [16,17,18] (Figure 2). The following section translates this multidimensional model into a staged research agenda.

## 6. Research Agenda for Multidimensional Acupuncture-Informed Neuromodulation

The proposed research agenda has three sequential objectives. Phase 1, characterization, defines and reproducibly manipulates the Baliao intervention space while identifying individual factors that influence anatomical access and proximal responses. Phase 2, mechanistic validation, identifies reliable measures of target engagement and tests whether these measures are linked to prespecified physiological functions. Phase 3, clinical translation, independently replicates the stimulation–engagement–function relationships before using them for patient stratification, parameter optimization, or adaptive stimulation. These phases need not be completed within a single study, but translational application should remain contingent on evidence generated in the preceding stages.

### 6.1. Phase 1: Characterization of the Intervention Space and Individual Context

Near-term studies should first define the dimensions of Baliao-region stimulation with sufficient precision to permit comparison and replication. Standardization in this context should not mean imposing a single fixed protocol. Rather, it should mean standardizing how the relevant dimensions of the intervention are described, measured, and varied. These dimensions include the stimulated location, laterality, modality, needle depth and trajectory, anatomical structures approached, electrical waveform, frequency, pulse width, intensity, stimulation duration, treatment schedule, and elicited sensory or muscular responses.

Surface-based localization should be supplemented, where feasible, by ultrasound, MRI, CT-based reconstruction, or three-dimensional sacral modeling to characterize posterior sacral foraminal anatomy, tissue depth, and individual variation in anatomical access [14,34,35]. These methods may help determine whether nominally identical Baliao protocols actually engage comparable tissue territories across participants. They may also clarify when different surface locations or trajectories converge on overlapping structures.

Participant-level factors should be characterized alongside intervention variables. Relevant modifiers may include body habitus, sacral morphology, baseline pelvic-floor activity, sensory thresholds, autonomic state, clinical phenotype, symptom severity, and previous treatment exposure. These variables should not initially be treated as predictors of treatment success by assumption. Their first role is to explain variability in tissue access, elicited responses, and physiological engagement.

Early parameter-mapping studies should use designs capable of separating the effects of individual intervention dimensions. Within-participant crossover, dose–response, and factorial designs may be especially informative for comparing depth, trajectory, modality, and electrical parameters while reducing between-person variability. The aim at this stage is not primarily to demonstrate clinical efficacy but to determine which combinations of intervention features produce consistent and distinguishable proximal responses.

### 6.2. Phase 2: Mechanistic Validation of Target Engagement and Functional Linkage

The next stage should identify measures that indicate whether relevant tissues, afferent pathways, or regulatory circuits have been engaged. Candidate measures include the distribution and quality of elicited sensations, visible or electromyographic pelvic-floor activity, sensory or motor thresholds, sacral- or pudendal-related reflex responses, evoked electrophysiological signals, and immediate changes in pelvic-organ function. A response should not be regarded as a target-engagement biomarker solely because it occurs during stimulation. Its value depends on whether it shows temporal proximity to stimulation, systematic sensitivity to intervention features, acceptable test–retest reliability, and biological relevance to the proposed pathway.

Proximal measures are prioritized during the initial validation stage because they are temporally closer to stimulation and are more directly sensitive to changes in depth, trajectory, intensity, and electrical parameters. They are therefore better suited to testing parameter-dependent recruitment, represented as Link 1 in the proposed model. By contrast, neuroimaging and autonomic variables reflect more distributed, multisynaptic, and state-dependent responses that may integrate target engagement with downstream regulation, expectancy, and other contextual influences. These distal measures remain important for evaluating broader functional consequences and the engagement–function relationship, represented as Link 2, but they are less suitable as sole evidence that a particular sacral tissue or pathway was recruited. This prioritization is therefore sequential and methodological, rather than a claim that distal biomarkers are intrinsically less valuable.

Because no single measure is likely to identify tissue or circuit recruitment unequivocally, convergent measures should be used where feasible. For example, a change in pelvic-floor electromyography may be more informative when interpreted together with stimulation depth, sensory distribution, reflex responses, and anatomical imaging. Similarly, autonomic measures and neuroimaging may provide important evidence of broader regulatory or supraspinal effects, but their distributed and context-sensitive nature makes them more appropriate as complementary measures of downstream engagement than as stand-alone indicators of local sacral target recruitment.

Comparison conditions should be selected according to competing recruitment models rather than point nomenclature or anatomical distance alone. Anatomy-guided Baliao stimulation might be compared with an adjacent lumbosacral condition differing in depth, trajectory, or expected tissue access; a superficial condition producing similar cutaneous sensation; or a parameter-matched condition differing in electrical dose. Such comparisons would not provide a simple binary test of whether Baliao is specific. Instead, they could determine whether changes in recruited tissues, sensory responses, or electrical parameters lead to systematic differences in proximal engagement.

Once reproducible engagement measures have been identified, studies should test whether they are linked to prespecified physiological functions. The functional outcomes should be selected according to the clinical phenotype. For stress urinary incontinence, relevant measures may include pelvic-floor electromyography, urethral closure function, continence-related motor control, and urinary leakage. For urgency or overactive bladder, bladder sensation, urodynamic variables, and sacral reflex excitability may be more appropriate. Postvoid residual volume and recovery of spontaneous voiding may be relevant to urinary retention, whereas anorectal manometry and continence-related outcomes may be prioritized for bowel dysfunction.

Clinical outcomes and target-engagement measures should remain conceptually distinct. Clinical improvement demonstrates benefit under a particular protocol, whereas an engagement measure helps explain the physiological process through which that benefit may have occurred. The strongest inference would arise when systematic variation in an intervention feature produces a reproducible proximal response, and that response is temporally and quantitatively associated with a prespecified organ-level or clinical outcome. Serial measurements during, immediately after, and across repeated stimulation sessions may help establish this sequence.

### 6.3. Phase 3: Independent Replication and Clinical Translation

Relationships identified in exploratory and mechanistic studies should be replicated in independent samples before they are incorporated into clinical decision-making. Multicenter studies will require harmonized localization procedures, reporting standards, practitioner training, physiological measurements, and prespecified analysis plans. Replication should address not only clinical outcomes but also the stability of intervention–engagement and engagement–function relationships. Negative or inconsistent findings should be reported because they may reveal that a proposed relationship is limited to a specific configuration, phenotype, or measurement context.

Patient stratification should follow the identification of reproducible sources of response variability. Stress urinary incontinence, overactive bladder, urinary retention, chronic pelvic pain, neurogenic lower urinary tract dysfunction, and anorectal disorders should not be treated as a single pelvic phenotype. Potential stratification variables may include anatomical access, baseline sensory and motor function, urodynamic phenotype, autonomic state, symptom topography, and early physiological response to stimulation. Multivariable models may then be used to examine how intervention features and individual modifiers interact, but predictive models should be evaluated prospectively and externally rather than developed and interpreted within the same sample.

Parameter optimization represents a subsequent translational step. Once intervention features have been linked reliably to target engagement and functional responses, the stimulation dose, depth, trajectory, or modality could be adjusted according to an individual’s anatomical or physiological profile. Biomarker-guided and adaptive stimulation should therefore be regarded as outcomes of a validated multidimensional model rather than as assumptions introduced at the outset [16,17,18]. Closed-loop approaches would be justified only when an early measurable response consistently predicts a later physiological or clinical benefit.

**Figure 3:**
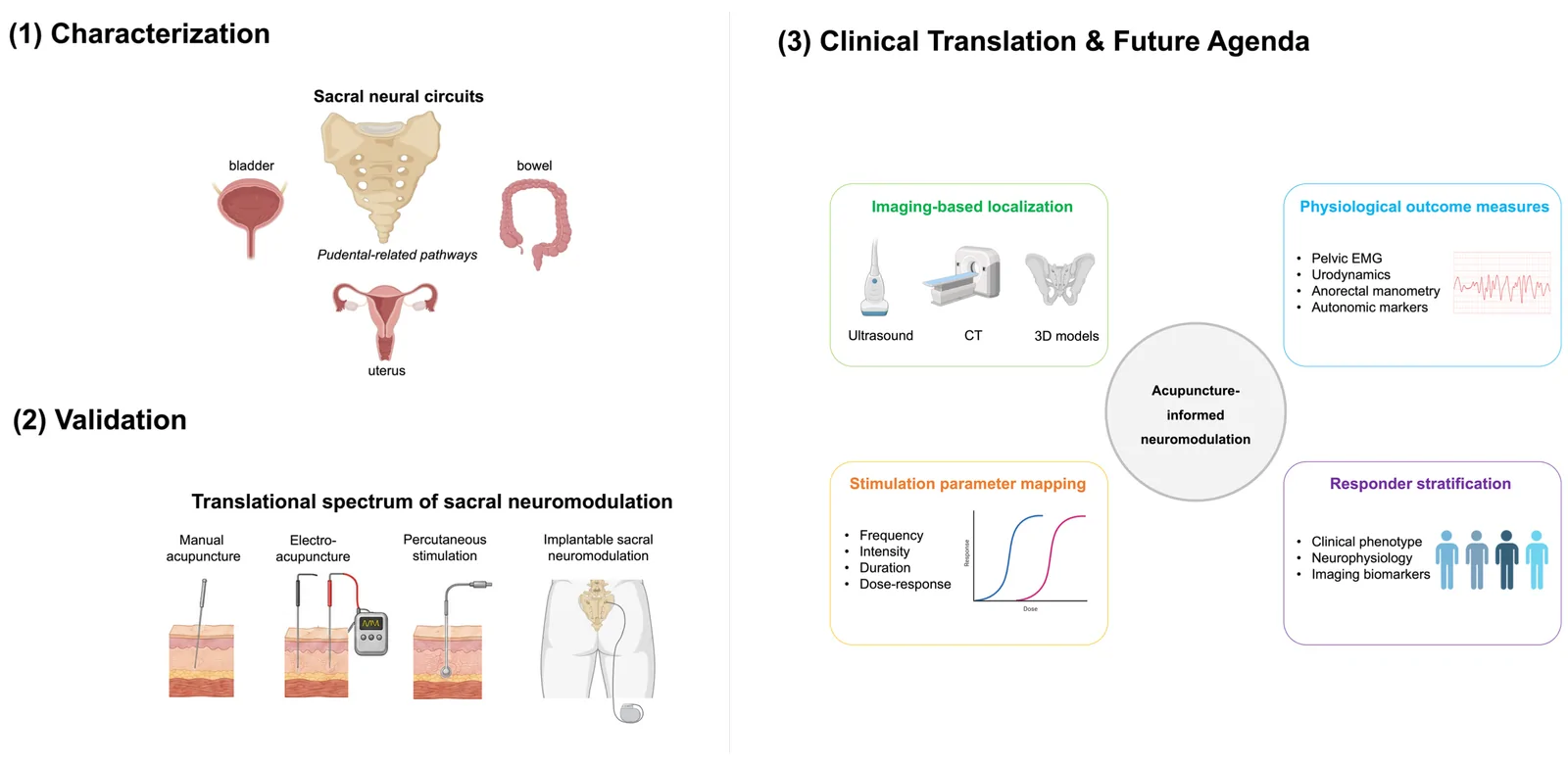
Sacral neural circuits, translational neuromodulation spectrum, and future research agenda for acupuncture-informed sacral neuromodulation. The upper-left panel summarizes sacral neural circuits related to pelvic organ regulation, including bladder, bowel, uterus, and pudendal-related pathways. The lower-left panel illustrates a translational spectrum of sacral neuromodulation approaches, ranging from manual acupuncture and electroacupuncture to percutaneous stimulation and implantable sacral neuromodulation. The right panel outlines key research directions for acupuncture-informed neuromodulation, including imaging-based localization, physiological outcome measures, stimulation parameter mapping, and responder stratification. Images used in the figure were created in Biorender and modified in Power Point www.biorender.com.

The framework should remain empirically revisable throughout this process. Support for a proposed interface would increase if defined combinations of intervention features produce reproducible engagement responses, if those responses are associated with prespecified physiological functions, and if the relationships replicate across independent samples. Conversely, failure to demonstrate parameter-dependent engagement, lack of association between engagement and function, or failure of replication should lead to modification of the proposed recruitment model, restriction of its functional scope, or rejection of the model for that indication. The purpose of this agenda is therefore not to confirm Baliao as a neuromodulation interface by assumption but to determine whether a historically defined intervention region can support reproducible, interpretable, and clinically relevant stimulation–response relationships (Figure 3).

## 7. Conclusions

Reframing acupoint and meridian systems as relational body maps offers a way to translate selected elements of classical acupuncture knowledge into testable neuromodulation models. In this framework, an acupoint-derived neuromodulation interface is defined not by a fixed point or presumed neural target but by the reproducible relationship between how a body region is stimulated, the biological processes it engages, and the functional responses that emerge within a given physiological context. The Baliao region illustrates both the potential and the current limitations of this approach. Its historically stabilized localization, sacral anatomy, and associations with pelvic function support its investigation as a candidate neuromodulation interface. However, current evidence does not establish a unique BL31–BL34 mechanism, absolute point specificity, or equivalence with implantable sacral neuromodulation. Progress therefore requires coordinated studies that systematically characterize stimulation variables and individual modifiers, identify reproducible measures of target engagement, and test their links to prespecified physiological and clinical responses. Although the framework may be extended to other acupoint regions, each region will require independent validation, while biomarker-guided, stratified, or adaptive stimulation should remain contingent on replicated stimulation–engagement–response relationships. Accordingly, the acupoint-as-interface framework should be regarded as a falsifiable working hypothesis and research agenda intended to generate prospective experimental studies, rather than as an already validated mechanistic model.

## Figures and Tables

**Table 1:** Illustrative acupoint regions and speculative candidate pathways for interface-based investigation.

Acupoint/Region	Classical Clinical Domain	Candidate Neuroanatomical Pathways	Measurable Physiological or Clinical Domain
Baliao BL31–BL34	Low back, pelvic, urinary, reproductive, and anorectal disorders	Sacral afferents, dorsal rami, pelvic floor-related pathways, autonomic reflexes, sacral spinal circuits	Sacral neuromodulation, pelvic organ control, urinary incontinence, overactive bladder, fecal incontinence, pelvic pain, and sacral reflex regulation
SP6 Sanyinjiao	Gynecological, urinary, pelvic, digestive, and reproductive disorders	Tibial and saphenous-region somatic afferents, lower-limb somato-autonomic reflexes, pelvic visceral regulation	Urinary symptoms, pelvic pain, dysmenorrhea, reproductive health, bladder sensory signaling, TRPV1/ATP-related mechanisms
ST36 Zusanli	Gastrointestinal regulation, fatigue, systemic weakness, immune regulation	Deep peroneal nerve region, somato-autonomic reflexes, vagal–adrenal or anti-inflammatory pathways	Gastrointestinal motility, inflammation, immune modulation, autonomic regulation
PC6 Neiguan	Chest discomfort, palpitations, nausea, vomiting, emotional distress	Median nerve afferents, cardiovagal/autonomic regulation, interoceptive pathways	Nausea/vomiting, heart-rate variability, autonomic modulation, perioperative symptoms
Auricular vagus-related points	Internal organ regulation, stress, pain, neuropsychiatric symptoms	Auricular branch of the vagus nerve, trigeminal and cervical auricular afferents	Transcutaneous auricular vagus nerve stimulation, autonomic regulation, inflammation, mood and pain modulation

## Data Availability

No new data were created or analyzed in this study. Data sharing is not applicable to this article.
